# GDI2 protein: research progress and its mechanisms in diseases

**DOI:** 10.3389/fcell.2026.1848213

**Published:** 2026-06-12

**Authors:** Changjian He, Tian He, Zan Zuo, Chao Yang, Yudan Zhang, Guanqi Su, Yunjiao Gong, Ping Wan, Wen Zhang

**Affiliations:** 1 Department of Gastroenterology, The First People’s Hospital of Yunnan Province, Kunming University of Science and Technology, Kunming, China; 2 Department of Gastroenterology, The First People’s Hospital of Yunnan Province, School of Medicine, Kunming University of Science and Technology Affiliated Hospital, Kunming, China; 3 Institute of Basic and Clinical Medicine, The First People’s Hospital of Yunnan Province, Kunming University of Science and Technology Affiliated Hospital, Kunming, China

**Keywords:** GDP dissociation inhibitor 2 (GDI2), rab GTPases, endocrine metabolism, cancer-related regulation, neurodegenerative diseases, infection immunity, pharmacological modulation

## Abstract

GDP dissociation inhibitor 2 (GDI2), also termed RabGDIβ, is an evolutionarily conserved Rab GDP dissociation inhibitor that is broadly expressed across species and is required for fundamental cellular functions. Mechanistically, GDI2 controls the cycling of Rab small GTPases between membranes and the cytosol, thereby regulating Rab-dependent membrane targeting, intracellular trafficking, and downstream signal transduction. Increasing evidence indicates that GDI2 is not only a constitutive component of the membrane-transport machinery; aberrant GDI2 expression or activity is associated with diverse human diseases. Notably, the functional consequences of GDI2 dysregulation appear to be context dependent, with reported involvement in cancer, neurodegeneration, immune dysregulation, and metabolic disease. Here, we synthesize current knowledge of GDI2 biology, highlighting recent advances that delineate its molecular features and its roles in maintaining membrane-trafficking homeostasis, remodeling signaling networks, and shaping disease-relevant cellular phenotypes. We also discuss the translational implications of GDI2 as a potential diagnostic biomarker and therapeutic target, and we propose a conceptual framework to guide mechanistic interpretation and the development of GDI2-directed therapeutic strategies.

## 1 Introduction

GDP dissociation inhibitors (GDIs) are essential chaperones that impart spatiotemporal control to small GTPase signaling. GDIs recognize and bind prenylated small GTPases, inhibit GDP dissociation, and extract them from membranes for recycling to the soluble cytosolic pool. Through these actions, GDIs sustain dynamic membrane–cytosol cycling of small GTPases. The GDI superfamily is commonly divided into three groups based on their preferred small GTPase clients and lipid-binding modes (i) RhoGDIs, which regulate Rho-family GTPases (e.g., RhoA, Rac1, and Cdc42): and thereby influence cytoskeletal organization and cell migration; (ii) RabGDIs, which act on Rab GTPases and are central to intracellular membrane trafficking, including vesicle budding, transport, and fusion; and (iii) phosphodiesterase δ (PDEδ), a GDI-like solubilizing factor that binds lipidated small GTPases (e.g., Ras) and modulates their spatial distribution (Cherfils and Zeghouf, 2013). Within the RabGDI subfamily, GDP dissociation inhibitor 1 (GDI1) and 2 (GDI2) are highly homologous and share core structural modules, yet they differ in tissue distribution, regulatory control, and physiological as well as pathological roles (Bächner et al., 1995). In humans, GDI1 is enriched in the nervous system and sensory tissues, whereas GDI2 is more ubiquitously expressed across tissues with limited tissue specificity (Fagerberg et al., 2013; Nishimura et al., 1994).

**FIGURE 1:**
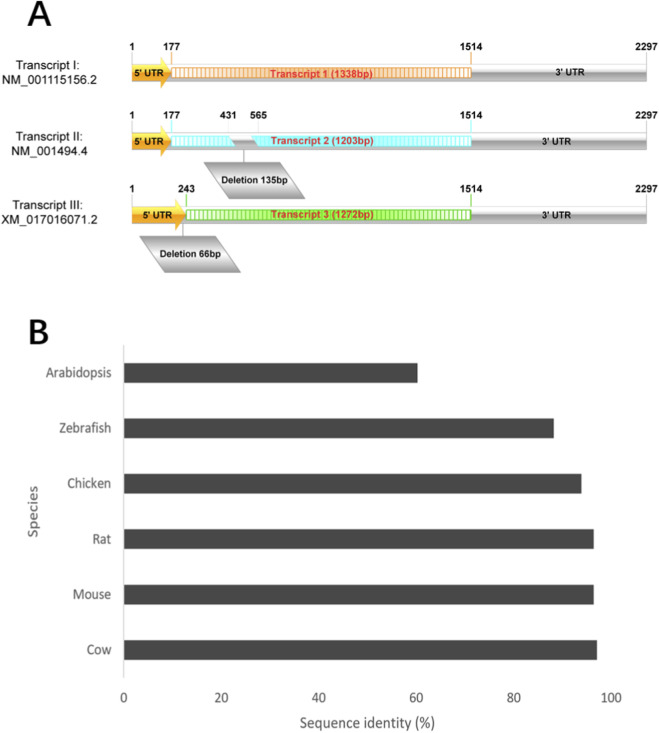
Structure and conservation of GDI2 genome. **(A)** Schematic overview of human GDI2 transcript variants. The human GDI2 gene produces three mRNA transcripts: transcript I (NM_001115156.2), transcript II (NM_001494.4), and transcript III (XM_017016071.2). Transcript I is the longest isoform (1,338 bp). Transcript II shares the same transcription start site as transcript I but lacks a 135 bp segment near the 5′untranslated region (1,203 bp). Transcript III initiates translation from an alternative downstream ATG located 66 bp from the canonical start codon (1,272 bp). The three transcripts are highly similar and differ mainly by segmental deletion and alternative translation initiation. **(B)** Evolutionary conservation of GDI2 across species. Amino acid sequence identity of representative GDI2 orthologs relative to human GDI2. Vertebrate species (cow, mouse, rat, chicken, and zebrafish) exhibit high sequence conservation (>88%), whereas the plant ortholog (*Arabidopsis thaliana*) shows lower sequence similarit.

The human GDI2 gene maps to chromosome 10p15 and spans approximately 50 kb. The gene contains 11 exons, and its introns are enriched for retrotransposon-derived elements. A processed pseudogene has also been reported at 7p11–p13 (Sedlacek et al., 1998). Notably, the human GDI2 locus generates three mRNA transcripts: transcript I (NM_001115156.2), transcript II (NM_001494.4), and transcript III (XM_017016071.2). The three transcripts exhibit high sequence similarity and differ primarily by segmental deletions or alternative translation initiation sites. As illustrated in Figure 1A, transcript I represents the longest and most complete isoform (1,338 bp). Transcript II shares the same transcription start site as transcript I but lacks a 135 bp segment near the 5′untranslated region, resulting in a total length of 1,203 bp. Transcript III differs from transcript I by initiating translation at an alternative downstream ATG located 66 bp from the canonical start codon, yielding a transcript length of 1,272 bp. These structural variations suggest potential regulatory diversity in GDI2 expression and may contribute to isoform-specific functional modulation.

GDI2 is a key chaperone that regulates the membrane–cytosol cycling of Rab small GTPases, which function as molecular switches controlling intracellular vesicular transport and membrane dynamics (Pfeffer et al., 1995). Rab proteins orchestrate multiple steps of membrane trafficking, including vesicle budding, transport, tethering, and fusion,and their activity depends on dynamic transitions between membrane-associated and cytosolic pools as well as between active GTP-bound and inactive GDP-bound states. (Homma et al., 2020). GDI2 preferentially binds GDP-bound Rab proteins, stabilizes the inactive conformation, prevents inappropriate nucleotide exchange, and promotes the extraction of membrane-associated Rab proteins into soluble cytosolic Rab–GDI complexes (Shisheva et al., 1999; Gavriljuk et al., 2013). This interaction limits Rab mislocalization and supports their redeployment to the appropriate membranes for subsequent trafficking cycles. Collectively, these actions help maintain the fidelity of Rab localization and sustain vesicular traffic across multiple routes, including endocytosis, recycling, secretion, and ER–Golgi transport (Stenmark, 2009; Hutagalung and Novick, 2011).

Overall, GDI2 is indispensable for Rab-mediated membrane trafficking. To assess evolutionary conservation, we compared GDI2 amino acid sequences across representative vertebrate and plant homologs. Relative to human GDI2, sequence identity was high among vertebrates: cattle (97.08%), mouse (96.40%), rat (96.40%), chicken (93.93%), and zebrafish (88.19%). In contrast, the *Arabidopsis thaliana* homolog showed substantially lower identity (60.23%). These data indicate that GDI2 is highly conserved across vertebrates and remains moderately conserved in distantly related eukaryotes, as displayed in Figure 1B.

## 2 Structure of GDI2

GDI2 is a 445–amino-acid protein with an estimated molecular mass of about 50.6 kDa (Shisheva et al., 1994). GDI2 is highly homologous to GDI1, and its core functional modules are conserved across the GDI family. Two sequence-conserved regions (SCRs)—SCR1 and SCR3B—fold together to form the Rab-binding site at the molecular apex. This site is a key structural determinant of Rab recognition and complex stability, enabling GDI2 to bind Rab GTPases preferentially in the inactive GDP-bound state. The architecture of this interface underlies the high-affinity interaction between GDI2 and Rab proteins. By stabilizing the GDP-bound conformation and limiting GDP dissociation, GDI2 maintains Rab in an inactive state and thereby regulates Rab cycling and vesicular trafficking (Shisheva et al., 1999). By contrast, differences between GDI2 and GDI1 are largely confined to variable C-terminal regions (residues 387–403 and 426–447). These regions likely have limited effects on Rab retrieval/solubilization *per se* but may contribute to regulatory functions or additional protein–protein interactions (Shisheva et al., 1995; Rivero et al., 2002), thereby conferring context-dependent specificity across cellular contexts and physiological or pathological states.

Although a high-resolution structure of GDI2 has not been reported, recent structural advances have refined our understanding of the GDI2–Rab interaction interface. Available evidence suggests that GDI2 can encapsulate Rab, shield the prenylated C terminus, and stabilize the GDP-bound conformation, thereby limiting nucleotide exchange and reducing Rab reassociation with membranes (Sun et al., 2023). Consistent with this model, GDI2 acts as a molecular chaperone that regulates Rab relocalization and membrane–cytosol cycling, providing a structural basis for its role as a spatiotemporal regulator of Rab signaling and vesicular trafficking.

## 3 Functions of GDI2

### 3.1 Functional basis of GDI2 as a regulator of Rab cycling

Rab proteins are among the principal molecular switches governing intracellular membrane trafficking. Their function depends not only on cycling between GDP- and GTP-bound states, but also on their dynamic partitioning between membrane-associated and cytosolic pools. Activated Rab GTPases are typically localized to defined membrane compartments, where they recruit downstream effector proteins to coordinate sequential trafficking events, including vesicle budding, transport, tethering, fusion, and cargo sorting (Wu and Chen, 2024). Upon completion of a trafficking event, Rab proteins undergo GTP hydrolysis and return to the GDP-bound state, after which they must be efficiently retrieved from membranes to enter subsequent rounds of cycling. Because most Rab proteins carry hydrophobic prenyl modifications at their C termini, once released from membranes they require dedicated chaperone proteins to remain soluble and competent for reuse (Edler and Stein, 2017).

GDI2 is a Rab GDP dissociation inhibitor that plays a central role in this recycling process. Unlike guanine nucleotide exchange factors, which directly promote Rab activation, GDI2 primarily recognizes and binds GDP-bound, prenylated Rab proteins, shields their hydrophobic lipid-modified C termini, and extracts them from membranes into the cytosol, thereby forming stable Rab–GDI2 complexes (Vélez-Reyes et al., 2021; Yin et al., 2024). Through this mechanism, GDI2 maintains a readily mobilizable cytosolic Rab reservoir while preventing the aberrant retention of inactive Rab proteins on membranes, thus ensuring their orderly redistribution among distinct membrane compartments. Subsequently, in response to local membrane cues and Rab activation machinery, Rab proteins can dissociate from GDI2 complexes, be retargeted to appropriate membrane domains, and enter a new round of functional cycling (Kim et al., 2019).

Accordingly, the function of GDI2 is not confined to a single Rab protein or an isolated trafficking route; rather, it provides a shared regulatory basis for sustained Rab turnover, spatial redistribution, and repeated reuse. When GDI2 activity is impaired, or when GDI2–Rab interactions are disrupted, the earliest consequences are often alterations in Rab membrane–cytosol balance and compartmental localization, which may subsequently translate into defects in specific membrane-trafficking steps (Sun et al., 2023; Maldonado et al., 2024). The following section discusses representative Rab proteins in greater detail to clarify the specific trafficking processes and functional consequences associated with GDI2 activity.

### 3.2 GDI2 in Rab-dependent intracellular trafficking pathways

Within the regulatory framework of Rab membrane–cytosol cycling described above, the function of GDI2 extends beyond the general recycling of Rab proteins. Through the compartment-specific roles of distinct Rab GTPases, this core mechanism is translated into several intracellular trafficking pathways with clearly differentiated biological functions. Available evidence suggests that GDI2-associated Rab proteins can be broadly grouped into several functional modules, including early secretory trafficking, endocytic sorting and receptor fate determination, cilium-related membrane transport, insulin-responsive GLUT4 trafficking, and the regulation of lipid-droplet homeostasis (Sun et al., 2023; Maldonado et al., 2024; Zhang et al., 2015; Chen et al., 2009; Guzmán-Ruiz et al., 2020).

In the early secretory pathway, Rab1A represents one of the best-characterized Rab proteins regulated by GDI2. Rab1A primarily coordinates trafficking among the endoplasmic reticulum, the ER–Golgi intermediate compartment, and the cis-Golgi, whereas GDI2 preserves Rab1A membrane–cytosol cycling and thereby supports its repeated reuse across these membrane compartments (Saraste, 2016). Available evidence indicates that disruption of the GDI2–Rab1A interaction impairs Rab1A retrieval from membranes and causes its aberrant retention in membrane fractions, which in turn blocks ER-to-Golgi transport (Sun et al., 2023). These findings underscore the importance of GDI2 activity in maintaining ER–Golgi trafficking flux and endomembrane homeostasis.

Within the endocytic system, GDI2 is functionally linked to Rab5/RAB5A, Rab7A, and Rab11A, which together shape cargo sorting and subsequent trafficking fate. Rab5/RAB5A operates at the early stage of this pathway, governing early endocytosis, early endosome formation and fusion, and the initial sorting of internalized cargo. After this early sorting step, cargo can proceed along two principal routes: one is directed by Rab7A toward late endosome maturation, multivesicular body–lysosome trafficking, and endolysosomal degradation; the other is mediated by Rab11A, allowing entry into recycling endosomes and subsequent redelivery to the plasma membrane (Kim et al., 2019). Accordingly, GDI2-associated regulation of the Rab5/Rab7A/Rab11A trafficking axis may influence the partitioning of endocytic cargo between degradative and recycling routes, thereby providing a trafficking basis for receptor transport, signal maintenance or termination, and the exploitation of host endosomal pathways by viruses during entry and post-entry trafficking (Yin et al., 2024; Bolado-Carra et al., 2021).

In the highly spatially organized context of cilium-related trafficking, Rab8/RAB8A represents another distinctive GDI2-associated regulatory axis. GDI2 maintains an inactive cytosolic Rab8GDP pool, thereby keeping Rab8 in a mobilizable state before its recruitment to cilium-associated membrane compartments. At the ciliary base, Dzip1 promotes the release of Rab8GDP from GDI2, enabling subsequent Rabin8-mediated activation and facilitating ciliary membrane assembly and ciliogenesis (Zhang et al., 2015). This process is functionally coupled to Rab11A: Rab11A-positive recycling vesicles deliver Rabin8 to the pericentrosomal region, creating the spatial conditions required for local Rab8 activation (Westlake et al., 2011; Knödler et al., 2010). Accordingly, the Rab11A–Rabin8–Rab8A axis illustrates a coordinated link between recycling endosome trafficking and ciliary membrane biogenesis.

GDI2 is also implicated in metabolism-related membrane trafficking. Rab4A is primarily associated with the early and recycling endosomal system, where it contributes to cargo recycling and endosomal resorting of GLUT4, thereby linking GDI2 activity to insulin-responsive recycling transport (Shisheva and Czech, 1997). Rab10 appears to act at a later stage, mainly regulating the movement, docking, and final delivery of GLUT4 storage vesicles to the plasma membrane. It should be noted, however, that current evidence more strongly supports a GDI1-biased role in Rab10 recycling, whereas GDI2 may exert auxiliary or context-dependent effects (Chen et al., 2009; Chen et al., 2012); by contrast, the functional association between Rab4A and GDI2 is more firmly established.

**FIGURE 2:**
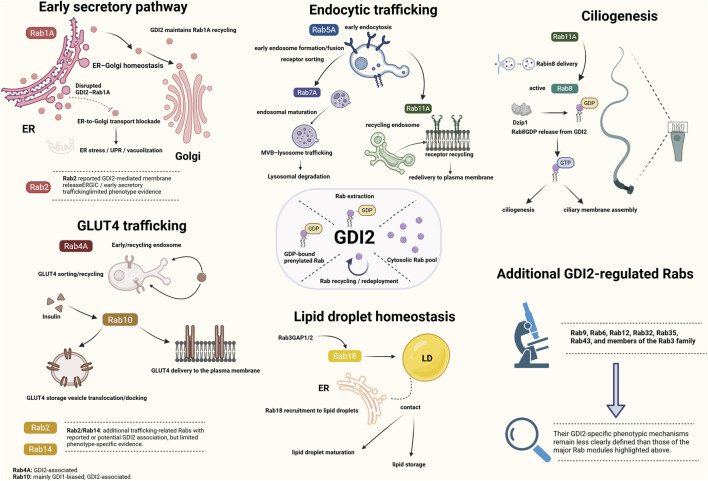
Schematic overview of representative Rab-dependent intracellular trafficking pathways associated with GDI2.

Distinct from the canonical vesicular trafficking routes discussed above, Rab18 extends the GDI2-associated Rab functional landscape to lipid droplet–endoplasmic reticulum contact-site biology. Upon activation, Rab18 is recruited to the lipid droplet surface and promotes endoplasmic reticulum–lipid droplet contacts, lipid droplet maturation, and lipid storage through NRZ–SNARE-related mechanisms. Available evidence further suggests that GDI2 can restrain Rab18 recruitment to lipid droplets, thereby modulating Rab18-dependent lipid droplet–associated trafficking processes (Guzmán-Ruiz et al., 2020; Xu et al., 2018). Beyond the representative Rab proteins discussed above, for which trafficking localization and mechanistic links to GDI2 are relatively well defined, additional studies have suggested that Rab2, Rab9, Rab6, Rab12, Rab32, Rab35, Rab43, and members of the Rab3 family may also be incorporated into the broader GDI2-regulated network (Shisheva et al., 1999; Yin et al., 2024; Nagai-Ito et al., 2022; Steger et al., 2017). Compared with the major functional modules outlined above, however, the GDI2-specific trafficking steps and phenotypic consequences associated with these Rab proteins have not yet been characterized with the same degree of mechanistic resolution. The representative GDI2-associated Rab proteins and trafficking modules are summarized in Figure 2.

GDI2 supports the reuse of distinct Rab proteins in specific trafficking pathways by maintaining the membrane–cytosol cycling of GDP-bound, prenylated Rab GTPases. The figure summarizes the major GDI2-associated Rab modules discussed in this review: Rab1A mediates early secretory trafficking; Rab5A, Rab7A, and Rab11A participate in endocytic sorting, degradation, and recycling; Rab8 together with the Rab11A–Rabin8 axis regulates ciliogenesis; Rab4A and Rab10 are involved in GLUT4 trafficking; and Rab18 contributes to lipid droplet–endoplasmic reticulum contacts and lipid droplet homeostasis. Rab2, Rab9, Rab6, Rab12, Rab32, Rab35, Rab43, and members of the Rab3 family may also be incorporated into broader GDI2/GDI-regulated networks, although their specific mechanisms remain to be further clarified.

## 4 GDI2 and diseases

### 4.1 GDI2 in endocrine regulation and metabolism

In endocrine and metabolic contexts, GDI2 modulates Rab-dependent vesicular trafficking, thereby influencing insulin-responsive cargo transport and lipid homeostasis and contributing to cellular metabolic adaptation. In adipocytes, accumulation of membrane-associated GDIs can impair insulin-dependent trafficking. The cellular redox state rapidly alters the membrane–cytosol distribution of GDI1/2. Reducing conditions promote GDI1/2 retention on membranes and nearly abolish insulin-stimulated translocation of glucose transporter 4 (GLUT4) to the plasma membrane, whereas hydrogen peroxide (H_2_O_2_) decreases membrane-associated GDI1/2 (Chinni et al., 1998). GDI1 and GDI2 colocalize with GLUT4 and Rab10 at the trans-Golgi network (TGN) and peripheral structures but differ in Rab selectivity. GDI1 shows higher affinity for Rab10, whereas GDI2 preferentially associates with Rab4A; overexpression of either isoform markedly suppresses insulin-stimulated GLUT4 translocation (Chen et al., 2009). Consistently, in 3T3-L1 adipocytes, Rab4 forms cytosolic complexes with both GDIs, and the GDI2–Rab4 interaction is regulated by tyrosine phosphorylation. These findings suggest that GDI2 may act in endocytic and recycling steps in a signal-tunable manner, thereby modulating insulin-responsive trafficking (Shisheva et al., 1995; Guzmán-Ruiz et al., 2020).

In a disease-relevant setting, GDI2 is selectively upregulated in omental adipose tissue from obese individuals with insulin resistance and can inhibit insulin-stimulated Rab18 association with lipid droplets, potentially perturbing lipid-droplet dynamics and lipid storage. Under insulin resistance–like conditions (high glucose/high insulin), increased GDI2 levels accompanied by reduced Rab18 localization to lipid droplets have also been reported (Shisheva and Czech, 1997). Beyond peripheral insulin action, integrative analyses combining genetic data with β-cell interaction networks have prioritized GDI2 in modules linked to GLP-1–promoted insulin secretion, implicating connectivity to a Rap1-centered node. Moreover, the rs871748 locus shows nominal associations with multiple insulin secretion phenotypes, raising the possibility that GDI2 contributes to trafficking control during secretion (Gudmundsdottir et al., 2018).

A study in HepG2 cells reported that a methanolic extract of tamarind (*Tamarindus indica*) pulp altered the secreted protein profile, including GDI2. The authors inferred potential effects on cholesterol metabolism, possibly involving LXR/RXR-associated cholesterol efflux pathways (Chong et al., 2012).

### 4.2 GDI2 in cancer

GDI2 has been implicated in tumor initiation, progression, and clinical outcomes across multiple cancer types; however, its functional effects are highly context dependent and may be bidirectional. Overall, elevated GDI2 expression has been reported in many solid tumors and is frequently associated with aggressive clinicopathologic features and adverse prognosis. For example, in hepatocellular carcinoma, GDI2 expression is higher in tumor than in non-tumor tissue (Yang et al., 2021; Ye et al., 2024), and elevated levels correlate with advanced stage, higher grade, and poorer survival. A similar association between high GDI2 expression and malignant progression has been reported in gastric, colorectal, oral squamous cell, thyroid, and esophageal squamous cell cancers, as well as glioblastoma and pancreatic cancer (Wang et al., 2025; Bai et al., 2010; Sun et al., 2007; Kashyap et al., 2010; Yin J. et al., 2025; Lo et al., 2006), often accompanied by increased proliferative, migratory, and invasive phenotypes. Conversely, in specific tumor types or molecular subtypes, several studies suggest that GDI2 may be associated with favorable outcomes and could exert metastasis-suppressive effects. For instance, proteomics-based association analyses in HER2-positive and ER-positive breast cancer indicated that higher GDI2 expression is associated with improved overall survival and a lower risk of disease progression (Wang Y. et al., 2024; Ren et al., 2023). In muscle-invasive bladder cancer and ovarian cancer, lower GDI2 expression has been linked to metastasis and poorer outcomes (Lee et al., 2010).

Emerging evidence indicates that GDI2 functions extend beyond basal membrane-transport regulation. In distinct oncologic contexts, GDI2 may influence cancer cell invasiveness and the immune microenvironment by altering membrane-trafficking homeostasis, receptor endocytic routing, and cytoskeletal or secretory programs. Collectively, current evidence supports two interrelated dimensions through which GDI2 may act in cancer.

#### 4.2.1 The Rab-mediated membrane-trafficking homeostasis axis

Mechanistically, GDI2 preferentially binds GDP-bound Rab proteins and facilitates their extraction from membranes into the soluble cytosolic pool. In doing so, GDI2 maintains Rab membrane–cytosol cycling and thereby helps organize vesicular transport and endocytic pathways in a spatiotemporally controlled manner (Ou et al., 2024; Nowak et al., 2022; de Graauw et al., 2013).

In HER2-positive trastuzumab-sensitive and trastuzumab-resistant breast cancer cell models, binding of the invasion-associated integrin αVβ6 to latency-associated peptide (LAP) triggers αVβ6 endocytosis and coincides with intracellular trafficking of human epidermal growth factor receptor 2 (HER2/ERBB2). HER2 subsequently traffics through RAB5A-positive early endosomes and then RAB7A-positive late endosomal compartments, which amplifies MAPK and Akt signaling and promotes tumor invasion. In this setting, GDI2 can restrain RAB5-dependent endocytic activity, thereby limiting excessive HER2 internalization and helping preserve plasma-membrane HER2. Notably, siRNA-mediated GDI2 knockdown in trastuzumab-sensitive cells dysregulates RAB5 activity, sustains HER2 endocytosis, reduces αVβ6 dependence of transforming growth factor-β (TGF-β) activation, and further enhances breast cancer cell invasiveness (Maldonado et al., 2024). Moreover, in breast cancer cells, the HMG-CoA reductase inhibitor lovastatin suppresses RhoA membrane translocation and reduces the membrane-associated active pool. Concomitantly, lovastatin upregulates GDI2, stabilizes RhoA in an inactive state, and reduces invasiveness (Klawitter et al., 2010).

When GDI2 is reduced or inhibited, Rab-dependent trafficking homeostasis is often disrupted early. Studies in hepatocellular carcinoma based on analyses of TCGA-LIHC patient data, paired tumor and non-tumor tissue comparisons, and expression validation in HCC cell lines suggest that GDI2 may influence the tumor microenvironment by modulating trafficking pathways that support extracellular matrix (ECM) remodeling and lipid metabolic programs (Zhang et al., 2021). Ding K and colleagues proposed that GDI2 may indirectly influence telomere length through effects on endoplasmic reticulum (ER) function, with downstream consequences for nucleotide metabolism, cell-cycle control, and oxidative or inflammatory stress responses (Ding et al., 2024).

In colorectal cancer cell models, GDI2 silencing suppresses proliferation and induces G0/G1 arrest, accompanied by activation of the p53 pathway, including increased phosphorylated p53 (p-p53) and phosphorylated p21 (p-p21). Mechanistically, GDI2 can interact with RAB5A, and RAB5A activity is inversely associated with p53 pathway activation (Ou et al., 2024). Notably, the GDI2 inhibitor BQZ-485 disrupts the GDI2–Rab1A interaction, blocks Rab1A recycling, and causes aberrant Rab1A accumulation on membranes. This mislocalization further impairs ER-to-Golgi transport, triggering ER expansion and fusion, ER stress, and the unfolded protein response (UPR), accompanied by cytoplasmic vacuolization and other paraptosis-like changes (Sun et al., 2023) (Ye et al., 2024; Ding et al., 2024).

From a therapeutic perspective, tumor cells—owing to high protein synthesis demand and elevated trafficking activity—may be particularly dependent on membrane-trafficking homeostasis. Accordingly, targeting the GDI2–Rab1A axis may preferentially compromise tumor cells while sparing normal cells. In prostate cancer, GDI2 is reported to be highly expressed and to promote tumor growth, at least in part by suppressing p75 neurotrophin receptor (p75NTR) signaling. These effects were evaluated using prostate cancer cell models and nude-mouse xenografts, with GDI2 knockdown used to assess its functional contribution. Paclitaxel has been shown to downregulate GDI2, restore p75NTR signaling, and thereby inhibit proliferation and promote apoptosis (Liu et al., 2021). By maintaining Rab membrane–cytosol cycling and trafficking homeostasis, GDI2 can shape endocytic routing of key receptors and downstream signaling outputs, positioning it as a nodal regulator of invasion, growth, and stress adaptation across tumor contexts.

#### 4.2.2 GDI2-driven migration/invasion and tumor microenvironment remodeling

Collectively, GDI2 may function as a regulatory node in small GTPase signaling networks and promote tumor cell migration and invasion by modulating focal adhesion turnover and actin cytoskeletal remodeling. In parallel, GDI2 may influence immune-cell recruitment by altering extracellular matrix (ECM) composition and chemotactic or inflammatory signaling, thereby contributing to tumor dissemination and tumor–stroma–driven remodeling of the tumor microenvironment (Lee et al., 2010; Lee et al., 2011).

In bladder cancer models, re-expression of RhoGDI2 markedly suppresses lung metastasis and reduces tumor-cell secretion of versican (VCAN). Reduced versican in metastatic lesions coincides with lower macrophage infiltration and diminished pro-inflammatory signals, including interleukin-6 (IL-6) and C-C motif chemokine ligand 2 (CCL2), suggesting that RhoGDI2 attenuates a metastasis-supportive inflammatory niche (Said et al., 2012). In our prior analysis of a hepatocellular carcinoma cohort, GDI2 was upregulated, and higher expression correlated with more aggressive clinicopathologic features and poorer prognosis. In LIHC cell models, GDI2 knockdown suppressed proliferation, migration, and invasion, consistent with a pro-tumorigenic role (Zhang et al., 2021). Moreover, GDI2 expression is associated with immune infiltration patterns: it correlates positively with T helper, Th2, and central memory T (Tcm) cells but negatively with cytotoxic cells, dendritic/plasmacytoid dendritic cells (DC/pDC), Th17 cells, B cells, and neutrophils, suggesting that GDI2 may shape the tumor microenvironment through immunologic remodeling (Ye et al., 2024; Zhang et al., 2021).

Beyond tumor cell–intrinsic programs, tumor–stroma interactions can regulate migration through chemotactic cues. In hepatocellular carcinoma, tumor-derived autocrine motility factor (AMF) enhances recruitment of mesenchymal stem cells (MSCs) to HCC lesions. In studies using human MSCs, recombinant AMF (rAMF) stimulation downregulated GDI2 mRNA, potentially relieving constraints on small GTPase activity to facilitate cytoskeletal reorganization and migration. rAMF also alters expression of the AMF receptor (AMFR) and caveolin-1/2, suggesting that GDI2 downregulation—together with coordinated transcriptional changes—supports directed MSC migration toward the tumor microenvironment (Bayo et al., 2014).

Additional studies across tumor types support an association between GDI2 and invasive phenotypes. In cutaneous squamous cell carcinoma, studies using stable K8-knockdown A431 skin carcinoma cells showed that keratin 8 (K8) knockdown is accompanied by reduced GDI2 expression and decreased Rac activity, with consequent attenuation of cytoskeletal remodeling and migratory capacity (Tiwari et al., 2018). In medulloblastoma, ectopic GDI2 expression in Shh-induced mouse medulloblastoma models promoted leptomeningeal dissemination, and complementary overexpression studies in cultured medulloblastoma-related cell models showed increased migration, invasion, and anchorage-independent growth, potentially by altering Rab trafficking and subcellular localization (Jenkins et al., 2014). In thyroid cancer, GDI2 is upregulated and shows concordant changes with cytoskeletal regulators, including destrin and stathmin (Onda et al., 2004). Upstream, microRNA-15b-5p (miR-15b-5p) directly targets the 3′-UTR of GDI2 and suppresses its expression. In thyroid carcinoma cell models, rescue experiments using GDI2 overexpression or si-GDI2 further showed that this miR-15b-5p/GDI2 axis modulates vesicle trafficking and the secretion or localization of matrix metalloproteinases (MMPs). This is accompanied by reduced MMP2 and MMP9 levels and ultimately suppresses thyroid cancer cell migration and invasion (Onda et al., 2004; Zou et al., 2020).

**FIGURE 3:**
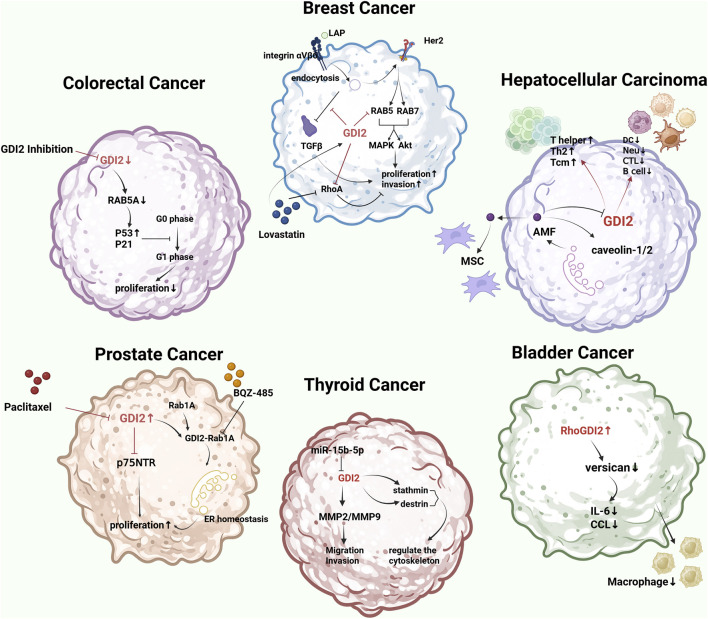
GDI2-mediated membrane trafficking, signaling regulation, and tumor microenvironment remodeling in various cancer types.

By coordinating small GTPase–dependent cytoskeletal remodeling and adhesion dynamics and linking these processes to ECM remodeling and immune-cell recruitment, GDI2 may collectively shape tumor invasive potential and microenvironmental states. To illustrate a framework in which GDI2 links Rab/Rho pathways to membrane trafficking, signaling outputs, and microenvironmental remodeling across tumor contexts, we synthesized the evidence and generated a schematic (Figure 3).

GDI2 exerts context-dependent regulatory effects on small guanosine triphosphatase (small GTPase) cycling, membrane trafficking, and downstream cellular programs across diverse tumor settings. By modulating the activation and subcellular localization of Rab and Rho family guanosine triphosphatases (GTPases), GDI2 influences receptor endocytosis and recycling, signaling persistence, cell-cycle control, cytoskeletal remodeling, and vesicle-dependent secretory processes.

### 4.3 GDI2 and other diseases

#### 4.3.1 GDI 2 and neurodegenerative pathology

A key pathological hallmark of Alzheimer’s disease (AD) is the generation and deposition of amyloid-β (Aβ) (Zheng and Wang, 2025). As a Rab GDP dissociation inhibitor, GDI2 regulates vesicular trafficking by controlling Rab GTPase membrane–cytosol cycling, thereby supporting neuronal endocytosis, secretion, and inter-organelle transport. Perturbation of this trafficking cycle may alter intracellular amyloid precursor protein (APP) routing and the subcellular milieu of APP processing, thereby shifting the balance between Aβ production and clearance.

Systems-level analyses of TMEM59-associated transcriptional regulatory networks have nominated GDI2 as a candidate AD gene, placing it within a module that links membrane trafficking to APP processing (Zhang et al., 2011). Functional data further support disease relevance: in 5xFAD mice with neuron-specific Gdi2 deletion, loss of GDI2improves learning and memory, reduces neuronal loss in the hippocampus and cortex, and decreases Aβ plaque burden and astrocyte activation, while increasing microglial activation and phagocytosis-related phenotypes (Wang M. et al., 2024). Mechanistically, in GDI2-knockout SH-SY5Y cells, GDI2 deficiency alters APP trafficking by increasing APP colocalization with the endoplasmic reticulum (ER) and reducing delivery to Golgi and endosomal compartments, which may reshape the APP-processing milieu and shift the Aβ production–clearance equilibrium (Wang M. et al., 2024). Plaque-adjacent proteomics in aducanumab-treated tgAPPPS1-21 mice further identified reduced GDI2 abundance, and other reports suggest altered GDI2 post-translational modification in AD brains (Bastrup et al., 2021). Together, these observations suggest that GDI2 may contribute to AD pathogenesis and could serve as a molecular correlate of local plaque remodeling and clearance after therapy.

The essential physiological role of GDI2 is supported by developmental and survival phenotypes: in GDI2-null mice, complete loss of GDI2 results in embryonic lethality. In Gdi2-deficient contexts, cell death is increased, as indicated by more TUNEL-positive cells; activation of apoptosis can be further supported by staining for cleaved caspase-3 (Wu et al., 2022a). During neurodevelopment and in mature neurons, membrane-trafficking homeostasis and apoptotic thresholds are inherently sensitive to disruption. When critical trafficking steps are compromised, transport defects can rapidly drive stress accumulation beyond a cellular tipping point, culminating in irreversible cell death (Wu et al., 2022b). Therefore, Rab–GDI2–mediated trafficking homeostasis is not merely housekeeping; it may instead underlie the particular vulnerability of cells with high trafficking demand, such as neurons, under degenerative stress (Han et al., 2025).

#### 4.3.2 GDI 2 and infection

GDI2 regulates Rab GTPase membrane–cytosol cycling and maintains trafficking homeostasis, thereby influencing immune-cell activation, secretion, and migration. Because antigen presentation, cytokine release, and motility depend on vesicular trafficking, GDI2 in macrophages and lymphocytes may help determine the magnitude and duration of immune responses.

Under homeostatic conditions, Siglec-family inhibitory receptors recruit phosphatases (e.g., SHP-1/2) via cytoplasmic immunoreceptor tyrosine-based inhibitory motifs (ITIMs), forming inhibitory signaling platforms that constrain excessive inflammation. Biochemical and cell-based interaction assays showed that GDI2 interact with the ITIM of sialic acid-binding immunoglobulin-like lectin G (Siglec-G), facilitating assembly of inhibitory signaling complexes and maintenance of immune tolerance (Wu and Chen, 2024; Wu et al., 2022b). Notably, after bacterial infection or inflammatory stimulation, the composition of ITIM-complex immunoprecipitates shifts: Rab1A is detected in the complex instead of GDI2, whereas under non-infected conditions GDI2 predominates over Rab1A (Wu et al., 2022c). This apparent replacement of GDI2 by Rab1A suggests that infection-induced complex remodeling may switch downstream ITIM regulatory modes and strengthen coupling between inhibitory receptor platforms and trafficking machinery.

During infection, GDI2 can also be regulated by metabolic signaling. Cell-based infection studies and mouse viral infection models showed that viral infection–induced itaconate production, itaconate can directly alkylate GDI2, weakening its ability to extract membrane-associated Rab GTPases into the cytosol and promoting Rab retention on membranes, thereby enhancing Rab-dependent viral entry and post-entry processes (Yin et al., 2024). Because many viruses remodel the host endomembrane system and build membrane-associated replication platforms, GDI2—positioned at a key checkpoint in Rab cycling—may be particularly susceptible to viral exploitation. For example, multiple CRISPR screens for severe acute respiratory syndrome coronavirus 2 (SARS-CoV-2) have repeatedly identified GDI2 as a host dependency factor. Protein interaction data further suggest that GDI2 interacts with multiple SARS-CoV-2 proteins, potentially contributing to membrane environments required for viral replication (Lata et al., 2021; Hasankhani et al., 2021).

**FIGURE 4:**
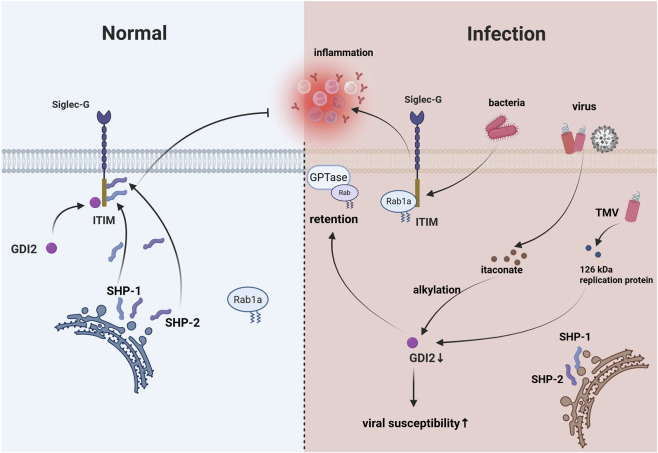
Different performance of GDI2-related immune responses to normal versus infectious status. Dynamic regulation of the Siglec-G–immunoreceptor tyrosine-based inhibitory motif (ITIM) inhibitory platform and Rab membrane cycling by GDI2 under homeostatic and infectious conditions: During bacterial inflammation, Ras-related protein Rab-1A (Rab1A) replaces GDI2 within the ITIM complex, whereas viral infection can induce inhibitory modification of GDI2, promoting Rab retention on membranes and thereby increasing viral susceptibility.

In plant TMV infection models, the 126-kDa replication protein has been reported to associate with host GDI2. Partial GDI2 silencing increases host susceptibility and is accompanied by vesicular rearrangements and altered GDI2 subcellular localization (Kramer et al., 2011). In mammalian systems, itaconate-mediated inhibition of GDI2 similarly promotes infection by vesicular stomatitis virus (VSV) and influenza A virus (IAV) (Yin et al., 2024). In summary, GDI2 is not merely a passive component of Rab cycling; it is a key node in immune homeostasis and infection responses that can be dynamically regulated by receptor signaling and metabolic pathways. Its functional state can influence assembly of inhibitory signaling platforms and may be exploited by pathogens to reprogram host membrane systems, thereby shaping infection outcomes. We summarize these contexts in Figure 4.

#### 4.3.3 GDI 2 and spermatogenesis/reproductive processes

Spermatogenesis, sperm maturation and capacitation, and early post-fertilization embryonic development depend on Rab-mediated vesicular trafficking and membrane-compartment dynamics. As a key chaperone in Rab cycling, GDI2 may participate in multiple reproduction-related processes.

Regarding sperm function and fertility, comparative proteomics of spermatozoa from high- and low-fertility crossbred bulls showed that Rab GDP dissociation inhibitor β (GDI2/RABGDI2) is upregulated in high-fertility groups and has been proposed as a potential biomarker of sperm function (Muhammad et al., 2018). In sperm transcriptomic analyses comparing fertile controls with asthenozoospermic and idiopathic normozoospermic infertile men, RABGDI2 was also identified among the upregulated transcripts. Moreover, co-expression network analyses connect RABGDI2 to genes involved in motility and cytoskeletal regulation, suggesting functional coupling to sperm motility phenotypes (Muhammad et al., 2018; Bansal et al., 2015).

Beyond sperm, Rab GDP dissociation inhibitor (RabGDI)–mediated Rab targeting and retrieval are also critical for early post-fertilization developmental programs. In *Arabidopsis thaliana*, interaction assays together with CRISPR/Cas9-generated GDI1 and GDI2 mutants showed that RabGDI interacts with multiple Rab GTPases and extracts Rab proteins from membranes after vesicle delivery to sustain Rab cycling. Simultaneous loss of GDI1 and GDI2 causes embryonic lethality, disrupts asymmetric zygotic division and polarity establishment, and is accompanied by defects in Rab5-associated trafficking (Yin G-M. et al., 2025). Proteomic profiling of bovine uterine luminal fluid during the pre-implantation period also indicates stage-dependent variation in GDI2. In bovine uterine luminal fluid, GDI2 is downregulated on day 16 of pregnancy relative to day 20, suggesting that it may contribute to a trafficking-related secretory microenvironment that changes with developmental stage (Forde et al., 2014). The disease-associated alterations, linked phenotypes, and potential mechanistic implications of GDI2 are summarized in Table 1.

**TABLE 1:** Disease-associated GDI2 alterations and their phenotypic and mechanistic implications.

Disease context	GDI2 alteration/Association	Linked phenotype	Potential mechanism	References
Colorectal cancer	GDI2 ↑ in CRC tissues and cells	GDI2 silencing suppresses proliferation, migration, invasion, and tumor growth; induces G0/G1 arrest	RAB5A–p53/p21 axis	Ou et al. (2024)
Hepatocellular carcinoma	GDI2 ↑ in HCC	GDI2 knockdown suppresses proliferation, migration, and invasion	Vesicle trafficking–related tumor progression and immune microenvironment remodeling	Ye et al. (2024), Zhang et al. (2021)
Prostate cancer	GDI2 ↑ in prostate cancer tissues	GDI2 silencing inhibits tumor growth and promotes cell-cycle arrest and apoptosis	Restoration of p75NTR-related signaling	Liu et al. (2021)
HER2-positive breast cancer	GDI2 participates in the αVβ6–HER2 trafficking network; recruitment ↓ in trastuzumab-resistant cells	HER2 internalization ↑ and invasiveness ↑ upon GDI2 dysregulation	RAB5/RAB7A/GDI2 trafficking subnetwork reshapes HER2 trafficking and signaling	Maldonado et al. (2024)
Pancreatic cancer	GDI2 ↑ in tumor tissues	GDI2 targeting induces paraptosis-like death and suppresses tumor growth	Disrupted GDI2–Rab1A interaction; blocked ER–Golgi trafficking; ER stress/UPR activation	Lee et al. (2010)
Thyroid cancer	GDI2 ↑ in tumor tissues	Proliferation and invasion ↑; miR-15b-5p tumor-suppressive effects weakened	Direct targeting of GDI2 by miR-15b-5p; sustained MMP2/MMP9 expression	Onda et al. (2004), Zou et al. (2020)
Medulloblastoma	GDI2 ↑ in medulloblastoma	Leptomeningeal dissemination and invasive phenotypes ↑	Enhanced invasion, migration, and anchorage-independent growth	Jenkins et al. (2014)
Obesity-associated insulin resistance	GDI2 ↑ in omental adipose tissue	Rab18 lipid-droplet recruitment ↓; lipid storage ↓	Restricted Rab18 association with lipid droplets; disrupted lipid-droplet homeostasis and adipocyte metabolism	Guzmán-Ruiz et al. (2020)
Alzheimer’s disease	GDI2 ↑ in 5xFAD mouse brains; Aβ induces neuronal GDI2 ↑	Neuron-specific Gdi2 deletion improves cognition and reduces neuropathological burden	APP trafficking to Golgi/endosomes ↓; APP processing and Aβ production ↓	Bastrup et al. (2021)
RNA viral infection (VSV/IAV models)	Itaconate alkylates GDI2 and impairs Rab extraction	GDI2 inhibition promotes VSV/IAV infection	Rab binding ↓; Rab membrane retention ↑; viral entry and post-entry trafficking ↑	Kramer et al. (2011)

CRC, colorectal cancer; HCC, hepatocellular carcinoma; VSV, vesicular stomatitis virus; IAV, influenza A virus; UPR, unfolded protein response. Mechanistic interpretations are based on the findings or proposals reported in the cited studies.

## 5 Pharmacological modulation of GDI2

From a pharmacological perspective, GDI2 is not merely a passive chaperone in vesicular trafficking; instead, it represents a functional node that can be modulated by diverse exogenous interventions operating at multiple level. The currently reported pharmacological strategies targeting GDI2, along with their molecular targets and downstream outcomes are summarized in Table 2. Generally, such interventions can be grouped into three categories. First, agents may act directly on GDI2 through covalent modification, reversible inhibition, or targeted degradation, thereby reducing its capacity to extract and recycle Rab proteins and altering Rab membrane–cytosol cycling. Second, GDI2 abundance may be modulated at the transcriptional or post-transcriptional level, with downstream consequences for tumor-associated phenotypes, including proliferation, apoptosis, invasion, and migration. Third, upstream signaling pathways, cellular redox status, or metabolic circuits may indirectly modulate the GDI2–Rab axis, converting perturbations in trafficking homeostasis into higher-order cellular responses, including stress signaling, rewiring of metabolic transport, or dysregulation of organelle-specific processes (e.g., ciliogenesis). Notably, GDI2-dependent outputs are strongly context dependent: perturbation of the GDI2–Rab cycle may facilitate pathogen exploitation of host membrane systems during infection, whereas in tumors it may expose trafficking vulnerabilities and elicit cell-fate changes.

**TABLE 2:** Summarizes currently reported pharmacological strategies targeting GDI2, along with their molecular targets and downstream outcomes.

Drug	Effect on GDI2	Mechanism of action / Target	Biological function	References
4-Octyl itaconate (OI)	↓	Alkylated GDI2 (Cys203/Cys335/Cys414)	VSV/IAV infection↑	Yin et al. (2024)
Itaconate	↓	Alkylated GDI2 (Cys203/Cys335/Cys414)	Viral infection/replication↑	Yin et al. (2024)
Paclitaxel (PTX)	↓	GDI2 mRNA↓ → p75NTR↑	G1 arrest→prostate cancer cells↓	Liu et al. (2021)
Cisplatin (DDP)	↓	GDI2 mRNA↓	proliferation in a subset of prostate cancer cells↓	Liu et al. (2021)
Carboplatin (CB)	↓	GDI2 mRNA↓	proliferation in a subset of prostate cancer cells↓	Liu et al. (2021)
BQZ-485	↓	Rab-binding platform (RBP)	Tumor cell apoptosis↑	Sun et al. (2023)
(+)-37	↓	Rab-binding platform (RBP)	Pancreatic tumor cells↓	Sun et al. (2023)
GDI2 degrader 21 (PROTAC)	↓	Ubiquitin–proteasome system	ER stress/UPR marker activation and vacuolization→tumor apoptosis↑	Sun et al. (2023)
N-acetyl-L-cysteine (NAC)	↓	ROS↓→reduced cellular state	Insulin-induced GLUT4 translocation to the plasma membrane↓	Chinni et al. (1998)
Pyrrolidinedithiocarbamate (PDTC)	↓	ROS↓→reduced cellular state	vesicular trafficking↓	Chinni et al. (1998)
Nordihydroguaiaretic acid (NDGA)	↓	ROS↓→reduced cellular state	insulin-mediated glucose transport signaling↓	Chinni et al. (1998)
Anti-Autocrine Motility Factor neutralizing antibody (anti-AMF Ab)	↓	MMP-2↑	Hepatocellular carcinoma cells↓	Bayo et al. (2014)
Aducanumab (anti–Amyloid-β antibody; human IgG1 isotype)	↓	Senile Plaque Penumbra Level 1 (SPP-L1)	Plaque burden↓; hippocampal senile plaques (SP)↓	Bastrup et al. (2021)
H₂O₂	↑	ROS↑→dissociation of GDI2 from the membrane	GLUT4 translocation to the plasma membrane↑	Chinni et al. (1998)
Tamarindus indica	↑	Not reported	Cholesterol transport and efflux↑	Chong et al. (2012)
Lovastatin	↑	Protein prenylation↓→GDI2mRNA↓	proliferation of breast cancer cells↓	Klawitter et al. (2010)

## 6 Conclusion

The strong evolutionary conservation of GDI2 across species underscores its essential role in maintaining cellular homeostasis. Prior studies have shown that GDI2 influences vesicular trafficking and intracellular signal transduction by regulating Rab GTPase cycling. However, GDI2 activity and molecular interactions vary markedly across cell types and disease states, and the determinants of this context dependence remain incompletely defined. This complexity likely reflects fine-tuned regulation of GDI2 within cellular microenvironments and highlights the need for integrative approaches that couple molecular and systems-level analyses. GDI2 is not merely a passive transport chaperone; instead, it functions as a regulatory switch within the intracellular membrane-trafficking system. Changes in GDI2 activity, abundance, or subcellular localization can first perturb Rab membrane–cytosol cycling and alter overall trafficking flux.

In cancer, the effects of GDI2 are context dependent and are shaped by its Rab clients, receptor sorting after endocytosis (recycling, degradation, or endosomal signaling), cellular dependence on trafficking and proteostasis, the integrity of tumor-suppressive networks (e.g., p53), and the cellular composition of the tumor. Consequently, GDI2 may function as a tumor promoter or suppressor depending on cancer type and molecular subtype. Mechanistically, GDI2 extracts GDP-bound Rab proteins from membranes into the cytosol, thereby maintaining a readily available cytosolic pool. Even within the same RabGDI2 framework, outcomes may diverge: GDI2 can either restrain endocytosis or sustain signaling, depending on receptor routing (Cherfils and Zeghouf, 2013). If receptors are recycled to the plasma membrane, signaling can be sustained and repeatedly reinitiated, increasing the likelihood of pro-tumorigenic output (Bächner et al., 1995). If receptors are routed to late endosomes/lysosomes, or if excessive endocytosis is restrained, signaling may be attenuated or terminated through degradation, which is more consistent with tumor-suppressive outcomes. This bidirectionality is further shaped by cellular context. In tumors with high trafficking demand and substantial secretory and protein-synthesis burdens, cells may be particularly dependent on stable ER–Golgi flux and membrane trafficking. Under these conditions, GDI2-mediated trafficking homeostasis can support growth and survival, whereas disruption of this process may compromise trafficking capacity and produce anti-tumor effects. In some models, GDI2 downregulation activates p53/p21 signaling and induces G0/G1 arrest. However, when p53 is deleted or inactivated, trafficking perturbations may no longer translate into cell-cycle restraint and may instead preferentially affect migration/invasion and stress adaptation. Nonetheless, key mechanistic aspects of how GDI2 regulates tumor biology remain unresolved and require further validation and refinement.

In immunity and infection, immune-cell functions depend heavily on membrane trafficking; antigen processing and presentation, receptor turnover, cytokine secretion, and chemotaxis all require vesicular transport. During infection, impaired GDI2-mediated Rab retrieval can increase membrane-associated Rab, potentially providing platforms that pathogens exploit for entry or replication. GDI2 may also contribute to inhibitory receptor platforms that maintain homeostatic suppression; however, infection and inflammation can remodel these complexes, and metabolic products can modify GDI2, thereby altering Rab membrane retention and trafficking efficiency.

Targeting GDI2 poses several practical challenges. First, GDI2 is a conserved GDI-family member that interfaces with multiple Rab proteins; thus, interventions are likely to produce cascading effects rather than modulating a single linear pathway. Pharmacological responses may therefore be non-linear and threshold dependent, with qualitative shifts emerging once perturbations exceed a critical level. Second, direct manipulation of GDI2 is likely to affect multiple trafficking routes, raising risks of tissue toxicity and unforeseen effects, particularly in immune contexts. Third, much of the current evidence is based on expression changes or proteomics associations, underscoring the need to distinguish causal mechanisms from correlative biomarkers when assessing whether GDI2 is a tractable therapeutic target. Overall, the field is moving beyond demonstrating pharmacologic modulability toward defining when modulation is safe and effective and identifying the diseases or molecular subtypes most likely to benefit.

At present, many conclusions rely on omics correlations, pathway enrichment, or network inference; the next step is to establish causal links with robust mechanistic evidence. For example, site-directed mutagenesis can test key post-translational modifications and binding interfaces; tracking complex assembly and disassembly can clarify regulatory logic; and live-cell imaging of Rab membrane retention, sorting trajectories, and trafficking flux can link trafficking changes to functional phenotypes.

From a translational standpoint, GDI2 may be better viewed as a network bottleneck than as a target within a single pathway. Indiscriminate inhibition or enhancement of GDI2 is likely to disrupt multiple fundamental trafficking processes. A more realistic strategy is to define the cell types and disease contexts in which GDI2 is outcome determining, identify the Rab subsets and trafficking steps most affected, and then develop context-specific interventions that minimize disruption of essential trafficking in normal tissues.

Overall, research on GDI2 is reframing membrane trafficking from a housekeeping process to a regulatable node with the capacity to influence disease outcomes. If future work provides systematic, mechanism-driven validation across immune, neural, and metabolic systems, it may clarify how homeostasis is disrupted and why such disruption translates into specific disease susceptibilities and clinical phenotypes. Multidisciplinary approaches will be essential to advance this field. Integrating molecular biology, genomics, proteomics, and clinical investigation should enable comprehensive mapping of the GDI2 regulatory network and identification of actionable intervention points. Emerging technologies, including single-cell profiling and high-resolution imaging, can further elucidate the spatiotemporal dynamics of GDI2. Together, these methodologies can bridge mechanistic discovery and clinical translation. In summary, continued investigation of GDI2 will advance understanding in cell biology and disease research. This work spans fundamental mechanisms and potential clinical applications, positioning GDI2 as a candidate therapeutic node for future intervention development. Realizing the translational potential of GDI2 will require sustained integration of diverse perspectives and technical approaches, with the goal of improving outcomes in diseases linked to aberrant GDI2 regulation.
